# The Gap Between the Actual Cost and Tariffs of Global Surgical Procedures: A Retrospective Cross-sectional Study in Qazvin Province, Iran

**DOI:** 10.34172/aim.31106

**Published:** 2024-10-01

**Authors:** Ehsan Zarei, Maedehsadat Hashemi, Pouria Farrokhi

**Affiliations:** ^1^Department of Health Service Management, School of Public Health and Safety, Shahid Beheshti University of Medical Sciences, Tehran, Iran; ^2^Department of Health Service Management, School of Public Health and Safety, Shahid Beheshti University of Medical Sciences, Tehran, Iran; ^3^Department of Health Management and Economics, School of Public Health, Tehran University of Medical Sciences, Tehran, Iran

**Keywords:** Global surgical procedures, Prospective payment system, Diagnosis-related groups, Reimbursement mechanisms, Hospital financial management

## Abstract

**Background:**

Iran’s healthcare system has a significant discrepancy between the national tariff and the cost of global surgical procedures (GSPs). This study aimed to compare the actual costs of GSPs with national tariffs in Iran’s public hospitals.

**Methods:**

This retrospective cross-sectional study was conducted in 2017. Using the census method, 6126 GSPs performed in three public hospitals were investigated in this study. Additionally, national tariffs from the Supreme Council of Health Insurance were obtained. The tariff-cost gap was the discrepancy between a GSP’s actual costs and tariff. Multiple linear regression analysis determined factors affecting the tariff-cost gap.

**Results:**

The average actual cost of GSPs was 637 USD, while the average tariff was 495 USD. The reimbursement covered only 78% of the costs. The gap was higher in older (B=1.05, 95% CI: 0.76-1.35, *P*<0.001), females (B=26.7, 95% CI: 15.5-37.9, *P*<0.001), patients with a longer stay (B=81.2, 95% CI: 77.5-84.8, *P*<0.001), and procedures performed by full-time surgeons (B=67.3, 95% CI: 56.9-77.5, *P*<0.001). Furthermore, neurosurgery had the highest effect on forecasting the gap between actual costs and tariffs among surgical specialties (B=346.9, 95% CI: 214.3-479.5, *P*<0.001).

**Conclusion:**

Public hospitals suffer from large financial losses due to the national tariff for many GSPs not covering their actual costs. It is suggested that tariffs be increased for certain customer segments that can bear higher costs and global tariffs be adjusted to match actual service delivery costs.

## Introduction

 Surgical procedures are a crucial, indispensable, and often costly aspect of public health in developed and developing nations,^1^ accounting for 30% of the global disease burden.^2^ Although surgical services and operating rooms are the main sources of hospital costs (approximately 40%), they generate the most revenue in most hospitals.^3^ Therefore, their reimbursement has attracted considerable attention. Surgical services in Iran are mainly provided in more than 1000 public and private hospitals.^4^ Government budgets, out-of-pocket payments, and health insurance are the main sources of financing,^5^ and the reimbursement mechanism for hospital services is mainly based on fee-for-service in Iran.^6^ Since 1999, a case-based payment system has been developed for 90 common global surgical procedures (GSPs), which is locally called “Global Payment”.^7^ Under this payment system, hospitals receive a fixed amount for each surgical procedure, according to the predetermined national tariff for that operation, which covers all costs such as the surgeon’s and anesthetist’s fees, medicine, operating room, and diagnostic tests.^8^ The amount of reimbursement is unaffected by factors such as gender, age, co-morbidity, and severity of the disease. In this payment system, the only variable that modifies expenses is the hospital’s level of accreditation, which has no bearing on other costs, such as surgeon fees.^8^

 The global payment imitates the diagnosis related groups (DRGs) payment system.^9^ This reimbursement mechanism is similar to “bundled payment” in America^10^ or the “payment by results” scheme in England.^11^ These payment mechanisms (including reimbursement for GSPs), which replace the traditional fee-for-service, reduce the incentive to increase the volume of services and costs.^10^ Moreover, they shift the risk from the payers to hospitals and patients and put the burden of cost minimization on hospital managers.^12^ The payment mechanism and tariffs are determined by the Ministry of Health.^13^ Under these arrangements, the provider is paid regardless of whether the treatments are provided. The flat fee incentivizes hospitals to maximize the number of procedures and reduce the cost per procedure. If the cost of surgery is more than the tariff it receives from the payer, the hospital loses part of its revenue. On the contrary, if a surgery costs lower than the tariff, a hospital can keep the extra money and use the profit.^11^

 However, the extent to which these tariffs reflect the actual cost of these surgeries has been questioned. Studies show that the actual costs are higher than the tariffs of common surgeries.^7,8,13^ This can lead to a loss and deficit for the hospital and negatively affect the quality of services, efficiency, and citizens’ access.^14^ This deficit must be compensated through government subsidies or out-of-pocket payments (formal or informal), which can expose patients to catastrophic costs.^15,16^ Globally and especially in developing countries, there is evidence that DRG reimbursement does not fully cover the costs of hospital services. For example, there is evidence from Greece that DRG reimbursement does not fully cover services such as neonatal intensive care unit care or cancer patients.^14,17^ Evidence from Switzerland in the treatment of burns,^18^ Italy in thyroid surgery,^19^ France for bariatric surgery,^20^ and the Czech Republic^21^ also indicate that the costs of care are not fully covered by insurance reimbursement.

 The reasonableness and applicability of medical tariffs are among the primary issues facing the Iranian healthcare system.^22^ Measures have been taken in recent years to solve the problem of unrealistic tariffs. In 2014, a series of reforms were made in the Iranian health system called the Health Transformation Plan. This reform was implemented in three phases to improve access to health care, financially protect households against healthcare costs (through reducing the amount of out-of-pocket payments), and finally achieve universal health coverage. The third phase of this reform included changes in medical tariffs and updating relative values.^23^ A study reported that medical tariffs doubled after this reform.^24^ Therefore, it was expected that these reforms would reduce the gap between the actual costs of GSPs and national tariffs.

 Little is known about the costs of surgical care and anesthesia in Iran. High-quality, standardized, and cost-effective analyses are needed to understand optimal platforms for surgical care delivery. Although studies have already been performed to determine the gap between tariffs and actual costs of GSPs in Iran, those studies have been either in a single surgery specialty or have been conducted in a single center.^7,8,13^ In addition, costs influenced by economic factors such as the inflation rate justify the necessity of such studies at different periods with up-to-date data to inform policymakers. Therefore, this study was performed to compare the actual costs of GSPs with national tariffs in Qazvin, Iran’s public hospitals.

## Materials and Methods

###  Study Setting and Design 

 This is a retrospective cross-sectional study conducted in Qazvin province, Iran, in 2017. Qazvin University of Medical Sciences covers 10 hospitals. Three hospitals were randomly selected due to limited resources. Two hospitals were teaching and tertiary hospitals (A = 255 beds and B = 230 beds), and one was a secondary and non-teaching hospital (C = 107 beds). Two researchers (MH and PF), who were completely conversant with the study’s objectives and the data collection process, gathered all the required data. All GSPs were reviewed from April 1, 2016 to March 30, 2017.

###  Population and Sampling 

 The target population included all patients who underwent one of the 90 GSPs. During the study period, there were 6,126 GSPs, all of whom were included in this study.

###  Data Collection

 The data source was the hospital information system, and the researchers were looking for the costs of the patients’ global treatment procedures in the review of the files. To avoid any errors and bias, all the data were entered into Excel software. The entries were double-checked to verify the accuracy and completeness of the collected data. The study data included the surgical procedure, the surgeon’s practice (full or part-time), the type of insurance, the length of stay (LOS), the patient’s gender and age, and the cost of the GSPs. In addition, GSP national tariffs were obtained from the Supreme Council of Health Insurance. GSPs were categorized into general surgery, obstetrics and gynecology, ophthalmic, orthopedics, urology, and neurosurgery groups.

###  Data Analysis

**Figure 1:**
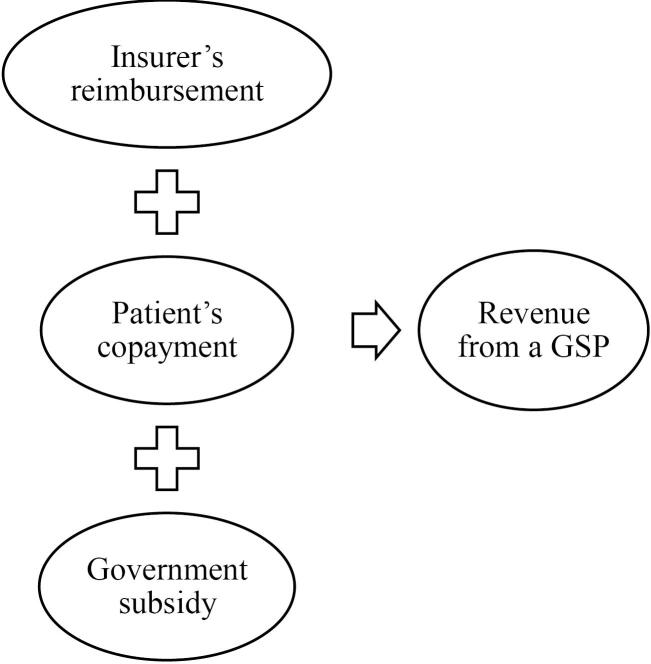
Components of Hospital Revenue From a GSP. *Note*. GSP: Global surgical procedure

 In this study, the actual cost was the cost recorded in the patient’s bill. The tariff-actual cost gap was defined as the difference between the average actual costs of a GSP and its tariff (positive or negative). Considering that government subsidies covered a portion of hospital expenses (to reduce patients’ out-of-pocket payments), the total amount of government subsidies plus patient copayments and insurer reimbursements was regarded as hospital revenue from a GSP (Figure 1). Tariff-revenue gaps were identified by comparing a GSP’s revenue with its national tariff and measuring the difference between both of them. Means, standard deviations, and frequencies were employed to describe the data. Multiple linear regression analysis was used in the analytical section to examine the relationship between the tariff-actual cost gap (as a dependent variable) and the independent variables (insurance type, surgeon practice type, patient age and gender, teaching status, LOS, and surgery group). The exchange rate of rials to USD was based on the data from the Central Bank of Iran.^25^ The obtained data were analyzed using IBM SPSS, version 22.

## Results

 Of the patients who underwent surgery, 53.6% were women, the average age was 39 (SD = 8.2) years, the Iranian Health Insurance scheme covered 65%, and the mean LOS was 2 ( ± 0.1) days. Most GSPs were related to general surgery (3,089 cases, Table 1), and the most common procedures were cataract (n = 774), vaginal delivery (n = 683), cholecystectomy (n = 567), inguinal hernia repair (n = 542), appendectomy (n = 540), cesarean section (n = 337), and hemorrhoidectomy (n = 330).

**Table 1:** Tariff, Actual Cost, and Revenue Profile of Common Surgical Procedures

**Surgical Category**	**Number**	**Average Tariff (USD)**	**Average Actual Cost (USD)**	**Average Revenue (USD)**	**Average Tariff-Actual Cost Gap (USD)**	**Average Revenue-Actual Cost Gap (USD)**
General surgery	3089	658	801	764	-143	-37
Gynecology and obstetric	1256	336	501	543	-165	+ 42
Ophthalmic	819	367	454	449	-87	-5
Orthopedic	540	208	427	367	-219	-60
Urology	414	387	442	475	-55	+ 33
Neurosurgery	8	852	861	1003	-9	+ 142
Total	6126	495	637	623	-142	-14

 The mean cost of GSPs was 637 USD, while the mean tariff was 495 USD (Table 1), indicating a notable negative gap between the national tariffs and the actual costs. In other words, 78% of the actual costs were covered by reimbursement. The costliest surgical procedure was femoral fracture fixation, with an average cost of 5764 USD, while the highest tariff was craniotomy, with 1020 USD.

 It was found that the average actual cost was between 0.1% and 29.4% lower than the tariff in nine cases. The greatest positive discrepancy between cost and tariff was observed in the case of nasolacrimal duct probing, with a cost-tariff gap of 29.4%. In 54 GSPs, the mean actual cost was found to be 0.3% to 307.4% higher than the tariff. The highest negative gap was found in the case of femoral fracture fixation, with a value of 307.4%. Conversely, the mean hospital revenue derived from each GSP was calculated to be 623 USD. This figure represents the sum of the insurer’s reimbursement, the patient’s copayment, and the government subsidy. When compared to the mean cost of GSPs, the revenue gap was determined to be -14 USD (Table 1).

 In 23 GSPs, the mean actual cost was between 0.1% and 33.3% lower than the revenue earned. The highest positive gap between the actual cost and revenue was related to the case of “femoral shaft fracture (open)”, with a value of 33.3%. The revenue generated from this surgical procedure exceeded its cost by 301 USD. In 40 GSPs, the mean actual cost was found to be 0.4% to 86.8% higher than the revenue earned. The highest negative cost-revenue gap belonged to the case of metacarpal amputation, with a value of -86.8%.

 The highest actual tariff-cost gap in GSPs was observed in the context of orthopedic operations, with an average loss of -219 USD (-52%) per procedure. Conversely, the lowest gap was identified in the domain of neurosurgeries, with an average loss of -9 USD (-1.1%) per procedure. Furthermore, the greatest gap between revenue and cost was related to the case of orthopedic operations, with a deficit of -60 USD per procedure. Conversely, for each neurosurgical procedure, the revenue generated exceeded the actual cost by an average of 142 USD (Table 1). The mean government subsidy paid for each obstetrics and gynecology procedure was 37 USD, representing the highest level of subsidy among the GSP groups. The lowest level of subsidy was found in the field of urology, with an average of 14.5 USD per surgical procedure.

 In fact, for each procedure, hospitals suffered an average of 142 USD losses (overall, 869 892 USD). However, when considering the revenues, the amount of the gap was reduced to 85 764 USD, and the loss per surgical procedure was 14 USD. A total of 143 290 USD in government subsidies was disbursed to hospitals for GSPs.

 A multiple linear regression analysis was conducted to examine factors affecting the gap between the tariffs and actual costs (Table 2). The analysis revealed that the overall results were statistically significant (F = 203.44, *P* < 0.001), and a robust relationship was observed between independent and dependent variables. The age and gender of the patients, the LOS, the surgical group, and the type of surgeon’s practice were found to significantly affect the tariff-actual cost gap. The gap was higher in older patients (B = 1.05, 95% CI: 0.76-1.35, *P* < 0.001), patients with a longer LOS (B = 81.2, 95% CI: 77.5-84.8, *P* < 0.001), females (B = 26.7, 95% CI: 15.5-37.9, *P* < 0.001), and procedures performed by full-time surgeons (B = 67.3, 95% CI: 56.9-77.5, *P* < 0.001). Moreover, neurosurgery had the most significant impact on predicting the gap between actual costs and tariffs across surgical specialties. The gap between the tariff and actual cost for neurosurgery procedures was statistically significant, with an average increase of 346.9 USD per procedure compared to orthopaedic surgeries (B = 346.9, 95% CI: 214.3 to 479.5, *P* < 0.001).

**Table 2:** Multiple Linear Regression Analysis of Factors Associated with the Tariff-Actual Cost Gap

**Variables**		**B **	**SE**	**[95% CI]**	***P***
Age		1.05	0.15	[1.35 to 0.76]	< 0.001
Length of stay		81.2	1.86	[84.8 to 77.5]	< 0.001
Gender	Male (Ref)				
	Female	26.7	5.71	[37.9 to 15.5]	< 0.001
Hospital type	Teaching (Ref)				
	Non-teaching	2.4	8.51	[-12.8 to 19.1]	0.771
Surgeon practice	Full time (Ref)				
	Part time	67.3	5.22	[56.9 to 77.5]	< 0.001
Insurance type	Social security (Ref)				
	Iranian health insurance	5.0	5.73	[-5.7 to 16.7]	0.334
	Relief committee	-9.5	19.2	[- 47.2 to 28.2]	0.625
	Armed forces	-9.3	13.8	[- 36.3 to 17.7]	0.501
Surgical specialty	Orthopedics (Ref)				
	Urology	186.3	12.6	[161.7 to 210.1]	< 0.001
	General surgery	147.4	9.12	[129.5 to 165.3]	< 0.001
	Ophthalmic	158.6	12.4	[134.3 to 182.8]	< 0.001
	Obstetrics and Gynecology	71.3	12.8	[46.2 to 96.3]	< 0.001
	Neurosurgery	346.9	67.6	[214.3 to 479.5]	< 0.001

*Note*. CI: Confidence interval; SE: Standard error.

## Discussion

 This study comparatively analyzed the actual costs of GSPs in Iranian public hospitals with their corresponding national tariffs. The findings revealed that the actual cost of GSPs was considerably higher than the amount reimbursed. Moreover, it was found that tariffs cover only 78% of the actual costs, leading to financial losses for hospitals. This can result in a deficit for the hospital and have a detrimental impact on the quality of services, efficiency of the hospital, an increase in out-of-pocket payments, and service accessibility.^14^

 Previous studies have shown that the current tariffs do not entirely cover the costs of GSPs in Iran.^7,8,13,26^ Aboutorabi et al calculated the difference to be $276, almost double our finding. This study was conducted only one year after our study, showing a sharp increase in costs and the stability of tariffs, which has led to a greater gap.^8^ Similarly, international studies have also reported comparable findings regarding reimbursement rates and actual costs. The findings of two studies in Greece about the DRG payment system for neonatal intensive care unit^14^ and cancer^17^ services demonstrated that the reimbursement rate was lower than the actual costs of these services. In a study conducted in France by Law-Ki et al, it was reported that for each “bioprosthetic abdominal wall reconstruction”, the hospital incurred a deficit of 15,233 euros. Furthermore, the reimbursement provided by the DRG system was insufficient to cover the total costs associated with the procedure.^20^ A study performed in Italy revealed that DRG tariffs were found to be lower than the actual cost of thyroidectomy.^19^ Varga et al in Hungary showed a considerable gap between the actual costs and the health insurance reimbursement for oesophageal cancer treatment.^27^ Additionally, the results of a study conducted in India represented that state-owned insurance reimbursed hospitals for numerous surgical procedures at significantly lower rates than the actual costs incurred.^28^

 The scenario in which the hospital’s cost for the operation exceeds the national tariff can be interpreted in two conflicting ways. Either the gap is due to inefficiencies, such as poor planning, long turnover, waste of resources, and the like, or it is an inherent problem with the tariff itself. Some common surgical procedures may be inherently unprofitable regardless of the level of efficiency achieved, even when performed with optimal efficiency. Conversely, other operations, such as cataracts, are likely to be profitable even if performed inefficiently.^11^ The calculation of national tariffs is so complex that it is susceptible to error and may impose a financial burden on hospitals in complicated cases or severe diseases because it does not adequately consider the additional costs caused by such cases.^29^ It would be reasonable to posit that the growth of medical tariffs should be proportional to the inflation rate.^22^ In recent years, there has been a notable increase in the prices of medicines and consumables, largely due to the significant rise in inflation. This has resulted in a corresponding rise in hospital costs. Conversely, the tariff rate has not increased in line with inflationary pressures.^30^

 Based on our findings, age, gender, LOS, and type of surgery were effective in the cost-tariff gap. In contrast, risk adjusters such as age, gender, disease severity, and co-morbidities are not considered in the GSP reimbursement.^7,8^ A number of studies have demonstrated that older patients are responsible for a greater proportion of service utilization and medical costs.^31,32^ Due to the presence of comorbidities or disease severity, older patients appear to utilize more resources, resulting in elevated costs.^32^ The data revealed a higher tariff-actual cost gap for surgical procedures performed on women. In general, women utilize healthcare services, and expenditure on these services is greater than that incurred by men.^33^ A study conducted by the Centers for Medicare and Medicaid Services demonstrated that $1.8 trillion was spent on healthcare for women in the United States in 2020, representing 54% of personal healthcare expenditure for women and 46% for men.^34^ A study performed in India reported that gender-specific conditions constituted 27% of hospital admissions and 15% of costs in women, a proportion that was higher than in men.^35^

 In our study, 20% of the GSP volume was attributed to obstetrics and gynecology, with these procedures representing the second-largest tariff-cost gap. As these surgical procedures are exclusively utilized for women, the type of service used by women is a contributing factor to the higher gap. In a Finnish study, 311 women receiving treatment for benign gynecological disorders had their medical records and expenses examined. It was shown that approximately half of hospital expenses were related to surgical procedures, with uterine fibroids and endometriosis treatments being the costliest.^36^ On the other side of the cost-tariff gap is the management of hospitals. The findings of a recent review indicated that Iranian hospitals are inefficient.^37^ This finding demonstrates that there is a waste of resources, and insurers’ reimbursements cannot cover the costs.^38^ In addition, the findings of a study revealed that after implementing the health transformation plan, there was a waste of resources in public hospitals.^39^ Therefore, hospitals should reduce costs by managing resource consumption and efficiency. Costs can be reduced by better planning operations (reduced cancellations, good scheduling for surgeries, and use of the full capacity of operating rooms) or emphasis on daycare (resulting in lower hospital stay costs). Advances and innovations in surgical procedures and new anesthetic techniques enable the transition to ambulatory settings. Ambulatory surgeries are generally less expensive because they require less staff, resource-intensive technologies, and infrastructure.^40^

 One of the key factors contributing to inefficiency and heightened costs is the prolongation of the patient’s hospitalization period. Inappropriate LOS in the hospital represents a significant source of inefficiency within the healthcare system.^41,42^ Patients who remain in the hospital for an extended period consume more hospital resources and generate greater costs.^43^

 The findings confirmed that the LOS was the most significant factor influencing the cost-tariff gap. For each additional day of LOS, the gap increased by 81.2 USD. A standard LOS has been specified for each procedure in the GSP reimbursement system. In the course of our study, it was found that the LOS of 15 GSPs was more than the standardized period. The standard LOS for metacarpal amputation is 1.8 days; in the present study, it was 3.5 days. It can thus be assumed that the extended LOS is a contributing factor to the observed increase in costs. The findings of a meta-analysis indicated that the DRG payment system has the potential to decrease costs by reducing the LOS.^44^ Nonetheless, reducing the number of unnecessary hospitalizations is one of the key challenges facing healthcare managers in increasing profits in prospective payment systems. Moreover, a study conducted in the United States demonstrated that reducing the length of hospitalizations can result in significant cost savings for healthcare institutions. The analysis yielded an estimated elasticity of patient LOS of 0.755, implying that longer stays are associated with higher costs.^45^

 One major factor contributing to the gap between actual and tariff costs was the expense associated with all surgical procedures. Neurosurgery procedure costs were the most significant factors influencing the difference between actual and tariffed costs among surgical procedures. A study analyzed the costs associated with neurosurgery procedures in a public hospital setting in the United States. It highlighted the substantial financial burden these procedures place on hospitals.^46^ Furthermore, a study conducted in a public teaching hospital in Iran revealed that the actual hospital bills for surgical procedures were considerably higher than the approved global tariffs. For instance, the cost of surgical procedures was determined to be 3%–312% higher than the approved global tariff in the majority of cases.^8^

 Teaching hospitals need a separate tariff as they often treat more complex patients and use more medical supplies and equipment because research and education are their primary goals.^8^ Further, patient co-morbidity is not considered in a tariff setting, leading to a longer patient stay and higher costs. In line with our results, a systematic review study in the UK indicated that co-morbidity (the presence of multiple chronic conditions) is associated with increased total healthcare costs, including hospital costs, hospitalizations, and emergency department visits.^47^ The failure to determine the actual tariff that covers the actual costs of the service can result in incomplete treatment and early discharge, which may in turn lead to increased surgical complications, re-admission rates, and treatment costs.^26^

 The results’ generalizability should be cautiously used due to the study’s limitations. The most important limitations are related to confounding variables. The first one is related to the patient’s demographics; age, gender, and comorbidities of patients can influence the cost of surgical procedures. For example, older patients or those with multiple health issues might require more resources, skewing cost comparisons. The second is the procedure complexity; the complexity and type of surgical procedures can widely vary, affecting costs. More complex surgeries typically incur higher costs, which might not be adequately reflected in national tariffs. The third is about the economic factors; inflation, currency fluctuations, and regional economic conditions can affect the costs of medical supplies and labor, leading to variations in actual costs compared to national tariffs. In addition, the performance of all members of the surgery team affects the result and ultimately the cost of the surgery. For instance, post-operative infection or other medical errors, which lead to a prolonged patient stay and an increase in the consumption of resources and ultimately an increase in the costs of hospitalization, may be due to the performance of the technicians rather than the performance of the surgeon. Information about comorbidities and surgical team members should be extracted from the patient’s medical record. Unfortunately, we did not have access to the patient’s medical records in this study. Therefore, the impact of comorbidity on costs should be considered in future studies. Moreover, our study was performed in three public hospitals in a province, and these hospitals may not have been representative of all public hospitals. For a deep understanding of the nature of the tariff-cost gap in GSPs, there is a need for large-scale studies with a larger number of hospitals in the country and a more detailed investigation of factors affecting this gap. Finally, for obtaining valid and reliable results, future studies should use strategies such as matching (individual and frequency matching), restriction, statistical control (e.g., multivariable regression analysis), and stratification to control confounding variables.

 Our study h­as important implications for researchers and policymakers working on reforming hospital payment systems:

- First, our findings align with existing literature on the under-reimbursement of surgical costs by insurance providers. Despite the modification of tariffs as part of Iran’s health reform, there remains a significant discrepancy between the reimbursed amount and the actual cost. The application of irrational tariffs presents a challenge to hospital administration. To maintain the motivation of healthcare providers, it is necessary to update national tariffs in line with the inflation rate. In the event of insurance failing to cover costs, patients may be required to make higher out-of-pocket payments. The imposition of user fees demonstrates a significant obstacle to patients seeking surgical and anesthetic care.
- The reimbursement mechanism is an effective tool for the financial management and control of hospital costs. The process of establishing tariffs should be conducted through negotiations between the health system authority, representatives of insurers, and representatives of healthcare providers. One of the key requirements for the Iranian health system is to transition from a global payment system for GSPs to a case payment system based on local DRGs.
- In a reimbursement system for GSPs, it is recommended that payments be adjusted based on a number of factors, including age, gender, co-morbidities, geographic location, and surgical method. In the absence of adequate risk adjustment, bundles may prove inequitable for healthcare providers. Such policies may even impede access to care for certain surgical candidates, as hospitals and physicians may be incentivized to prioritize patients with higher profit margins. It is imperative that more robust risk adjustment methodologies are employed to guarantee that healthcare providers are duly compensated and that patients are able to retain access to the care they require.
- It is suggested that GSP tariffs are differentiated for tertiary hospitals that admit complex cases. Given the educational function of these institutions, there is a potential for resource wastage. It is proposed that a base price for surgical procedures are established based on a calculation of actual costs, a relative weighting factor for the hospital level, and an additional payment for certain cases.
- The LOS of patients has been identified as a factor contributing to the cost-tariff gap. Consequently, the transfer of certain procedures to the outpatient and daycare settings has the potential to significantly reduce costs. Further studies are required to investigate this action in greater depth.

## Conclusion

 The findings of this study confirmed that the national tariff for numerous GSPs fails to align with the actual costs, resulting in considerable financial losses for public hospitals. It is recommended that GSP tariffs be revised to reflect the actual cost of hospital services. In a modified reimbursement system for GSPs, payments should be adjusted based on a number of factors, including age, gender, co-morbidities, geographic location, hospital level and function, and other risk adjusters.
